# Physical Impairments in People With Gout: A Scoping Review

**DOI:** 10.1002/msc.70103

**Published:** 2025-04-22

**Authors:** Pranav Kumar, Jenni Buckley, Edward Roddy, Martin J. Thomas

**Affiliations:** ^1^ School of Medicine Keele University Staffordshire UK; ^2^ School of Allied Health Professions and Pharmacy Keele University Staffordshire UK; ^3^ Centre for Musculoskeletal Health Research School of Medicine Keele University Staffordshire UK; ^4^ Haywood Academic Rheumatology Centre Midlands Partnership University NHS Foundation Trust Haywood Hospital Staffordshire UK

**Keywords:** exercise, gout, physical activity, physical impairment, physical intervention

## Abstract

**Introduction:**

Gout is the most common form of inflammatory arthritis. It is predominantly managed with pharmacological interventions, and physical impairments in people with gout have seldom been studied. We aimed to identify gout‐related physical impairments that may be targeted by physical interventions.

**Methods:**

Five electronic databases (Medline, AMED, EMBASE, APA PsycInfo, CINAHL) were searched from inception to April 2024, together with reference lists of all included articles. We included all study designs, except for singular case reports, conducted in people with gout, where at least one objective physical impairment outcome was reported. All title, abstract and full‐text article eligibility screening was performed independently by two reviewers. Independent data extraction included design and setting, participant demographics, baseline characteristics, disease duration, physical impairment investigated, and method of assessment. Data synthesis was summarised descriptively.

**Results:**

Twenty‐four articles were included. Most studies were cross‐sectional designs in secondary care settings, 11 were performed in New Zealand. Participants' mean ages ranged from 41.3 (standard deviation (SD) not calculated) to 75.8 (SD 5.2) years. Participants were predominantly male. Gout duration ranged from 24 h to a mean of 28 years. Five broad categories of physical impairment were identified: lower extremity function, joint range of motion, strength, deformity, and Achilles tendon stiffness.

**Conclusions:**

Based on limited evidence, the most commonly observed physical impairments are related to lower extremity function and joint range of motion. Our review identifies the need to better understand and quantify gout‐related physical impairments before developing targeted physical interventions.

## 1 Introduction

Gout is the most common inflammatory arthritis, affecting 3.2% of people in the UK in 2023 (Russell et al. 2023). It develops when elevated serum urate levels (hyperuricaemia) lead to the formation and deposition of monosodium urate (MSU) crystals in and around joints. Crystal deposition leads to recurrent severely painful inflammatory flares, tophus formation, and chronic arthropathy.

Management of gout comprises pharmacological strategies, both anti‐inflammatory treatment for flares and long‐term urate‐lowering therapy (ULT) to lower serum urate levels, prevent new crystal formation and bring about dissolution of existing crystals (Hui et al. 2017; Richette et al. 2017; FitzGerald et al. 2020; Neilson et al. 2022). Non‐pharmacological management focusses solely on dietary modifications to support weight loss or reduce consumption of food or drinks that may contribute to hyperuricaemia or trigger flares, such as red meat or alcoholic beverages (Choi et al. 2004a, 2004b; Zhang et al. 2012; Neogi et al. 2014). The possibility that people with gout could benefit from physical interventions such as exercises or orthoses has received little attention but is plausible. Gout predominantly affects the lower limb, with almost all people with gout experiencing foot involvement (Roddy 2011). Chronic gouty arthropathy is associated with chronic joint pain, tophi and joint damage and bone erosion (McCarthy et al. 1991; Sapsford et al. 2017). People with gout commonly have multiple comorbidities, including osteoarthritis (Roddy et al. 2007, 2008), which is associated with physical impairments such as muscle weakness (e.g. O'Reilly et al. 1998; Øiestad et al. 2022) and joint malalignment deformity (e.g. Sharma et al. 2010), for which non‐pharmacological modalities are a core component of management (National Institute for Health and Care Excellence 2022). However, there have been few studies on the extent to which people with gout have physical impairments that might be amenable to physical interventions. Small observational studies have reported muscle weakness, joint deformities, and reduced range of motion in people with gout (Stewart et al. 2015, 2016; Petty et al. 2019). People with tophaceous gout participating in a qualitative study described gout causing restricted range of joint motion and deformity, activity limitation and participation restriction (Aati et al. 2014). A small pilot study found that commercially available footwear reduced foot pain and disability in people with gout (Rome et al. 2013).

We undertook a scoping review to identify gout‐related physical impairments that may be targeted by physical interventions delivered in healthcare settings.

## 2 Methods

Our review was undertaken in accordance with the Joanna Briggs Institute methodological guidance (Peters et al. 2020), examining studies of people with gout (participants), who have physical impairments (concept) for which they could obtain treatment intervention in clinical healthcare settings (context). The design of our review is consistent with the Arksey and O'Malley framework, with the exception of the optional consultation exercise (Arksey and O'Malley 2005). The review was undertaken and reported in accordance with the Preferred Reporting Items for Systematic Reviews and Meta‐analysis Extension for Scoping Reviews (Tricco et al. 2018). Consistent with these guidelines, we did not appraise the quality of the included studies due to the range of methodological designs adopted.

### 2.1 Eligibility Criteria

We included all study designs across all care settings, with the exception of singular case reports, conducted in people with a gout diagnosis that included at least one objective physical impairment outcome. We excluded studies reporting only self‐reported physical impairment data, non‐human studies, editorials, review articles, and letters. There were no language restrictions.

### 2.2 Search Strategy

Five databases were searched using two database interfaces from inception to 11^th^ April 2024. Medline, AMED and EMBASE were searched using Ovid. APA PsycInfo and CINAHL Plus with Full Text were searched using EBSCO. A faculty research fellow assisted in refining the search strategy and helped develop keywords and optimise fields. The integrity of the strategy was verified by identifying the inclusion of a known study that met all the eligibility criteria. The full search strategy can be found in the Supporting Information S1. In addition, we screened the reference lists of all included studies for further eligible studies.

### 2.3 Study Selection

Retrieved articles from the five searched databases were exported into the Rayyan reference management software package (Ouzzani et al. 2016). Duplicates from across databases were removed by PK. Title, abstract and full‐text article eligibility screening was performed independently by PK and JB, with discrepancies resolved by ER and/or MJT.

### 2.4 Data Extraction and Synthesis

Data extraction was standardised using a data collection form and independently undertaken by two reviewers (PK and JB), with checking and arbitration performed by ER and/or MJT. Data extraction included design and setting, participant demographics, baseline characteristics, disease duration, physical impairment investigated, and method of impairment assessment. Where possible, comparative information about non‐gout and gout populations, or information about gout only populations, was extracted. The included studies were summarised descriptively and all extracted information from the data collection form was tabulated.

## 3 Results

### 3.1 Study Selection

**FIGURE 1:**
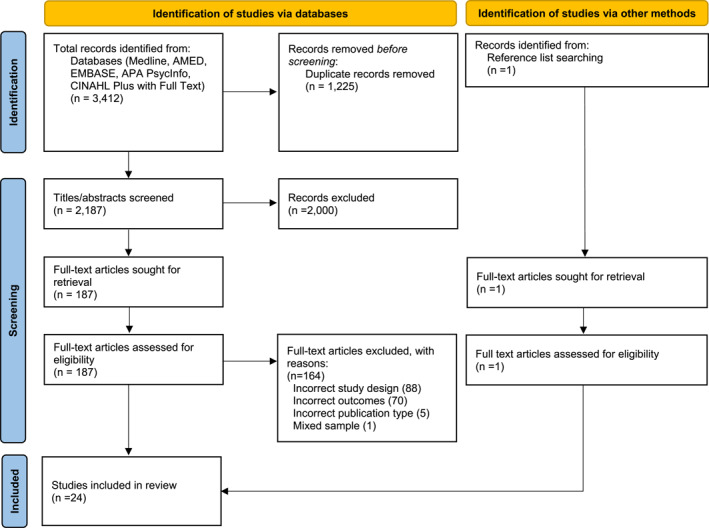
Study flow chart (adapted from Page et al. 2021).

In total, 3412 unique citations were identified from the database search (Figure 1). After duplicates were removed, 2187 titles and abstracts were screened, with 188 full‐text articles being assessed. Of these, 164 articles were excluded after full‐text review for the following reasons: incorrect study design, 88; no physical impairment outcomes, 70; incorrect publication type (single case reports, editorials, review articles, and letters), 5; and mixed sample of conditions without disaggregated gout data, 1. This resulted in 23 studies eligible for inclusion. One additional article was identified following screening of the reference lists of the included studies, resulting in 24 included studies.

### 3.2 Characteristics of Included Studies

Of the 24 included studies, 20 were undertaken in secondary care and one in primary care, two were community/population‐based studies, and for one study the setting was unclear (Table 1). All studies were published between 1991 and 2023. One study was translated from Chinese (Feng and Xiong 2021). Most studies were cross‐sectional (*n* = 17), followed by cohort studies (*n* = 5, one retrospective), then singular randomised controlled trial (*n* = 1) and case‐series designs (*n* = 1). Studies originated from eight countries, most commonly New Zealand (*n* = 11), followed by China (*n* = 3), United States (*n* = 3), Taiwan (*n* = 2), United Kingdom (*n* = 2), France (*n* = 1), Malaysia (*n* = 1), and Mexico (*n* = 1). The combined sample size of the included articles was 10,107, and the majority of study participants were male (range 60%–100%). Five study samples were exclusively male. Across the included studies, where reported, mean age ranged from 41.3 (standard deviation (SD) not calculated) to 75.8 (SD 5.2) years and gout duration from 24 h to a mean of 28 years. Overall study sample sizes ranged from 7 to 5819 participants. Studies examined physical impairments in the following areas of the body: foot/ankle (*n* = 7), hand/wrist (*n* = 4), knee (*n* = 3), lower extremity (*n* = 2) and multiple joints (*n* = 5). Joint location was unclear for three studies.

**TABLE 1:** Descriptive characteristics of included studies.

Author, year, country, setting	Study design	Gout diagnosis	Gout phenotype	Sample size	Age (years), mean (SD)^a^	Sex, % male	Ethnicity	BMI, kg/m^2^ Mean (SD)	Flare frequency	Tophi (%)	Gout duration (years), mean (SD)	Serum urate level, mean (SD)
Blandin et al. 2018, France, secondary care	Cross‐sectional study	MSU crystal identification	No specified phenotype	Total: 97 Gout: 56 Controls: 41	Gout: 63.9 (12.2) Controls: 59.0 (12.8)	Gout: 87.5 Controls: 90	NS	Gout: 27.3 (4.8) Controls: NS	NS	Gout: 27.7 Controls: N/A	Gout: 4.95 (5.2) Controls: N/A	Gout: 506.8 (111.1) μmol/L Controls: N/A
Burke et al. 2015, United States, Community	Cross‐sectional analysis in a prospective cohort study	Self‐reported physician diagnosis	No specified phenotype	Total: 5819 Gout: 595 No gout: 5224	Gout: 75.8 (5.2) No gout: 75.4 (5.1)	Gout: 60.0 No gout: 40.1	Gout: Black, 29.9% No gout: Black, 20.9%	Gout: 30.4 (5.7) No gout: 28.5 (5.6)	NS	NS	NS	Gout: 6.6 (1.9) mg/dL No gout: 5.7 (1.5) mg/dL
Carroll et al. 2018, New Zealand, secondary care	Cross‐sectional study	1977 American Rheumatism Association preliminary criteria (Wallace et al. 1977)	Tophaceous gout	Total: 48 Gout: 24 Controls: 24	Gout: 61.9 (12.0) Controls: 61.7 (12.3)	Gout: 92 Controls: 92	Gout: European: 58% Māori: 4% Pasifika: 25% Asian: 13% Controls: European: 96% Māori: 4% Pasifika: 0% Asian: 0%	Gout: 31.1 (4.1) Controls: 26.3 (5.1)	Gout: Number in last 3 months: **Mean (SD)** 1.23 (1.45) Controls: N/A	Gout: 100 Controls: N/A	Gout: 17.4 (11.9)	Gout: 0.37 (0.11) mmol/L Controls: N/A
Dalbeth et al. 2007, New Zealand, Secondary care	Cross‐sectional study	1977 American Rheumatism Association preliminary criteria (Wallace et al. 1977)	No specified phenotype	20	**Median (range)** 57.5 (38–75)	95	New Zealand European: 30% Pacific: 30% New Zealand Māori: 25% Indian: 10% Filipino: 5%	NS	NS	80	**Median (range)** 17 (1–50)	**Median (range)** 0.48 (0.25–0.68) mmol/L
Feng and Xiong 2021, China, secondary care	Retrospective cohort study (5 months‐9 years follow‐up)	ACR/EULAR classification criteria (Neogi et al. 2015)	No specified phenotype	24	**Median at baseline:** 50	88	NS	NS	NS	NS	**Range** 7 days–21 years	486.9 (135.0) μmol/L
López López et al. 2017, Mexico, secondary care	Two successive cross‐sectional studies	1977 American Rheumatism Association preliminary criteria (Wallace et al. 1977) & Clinical gout diagnosis (Vázquez‐Mellado et al. 2012)	No specified phenotype	Total: 564 Group A (1995–2000): 316 Group B (2010–14): 248	NS	Group A: 99 Group B: 97	NS	NS **Obesity** ^b^ **(%)** Group A: 62 Group B: 34	NS	Group A: 63 Group B: 67	Group A: 11.3 (9.0) Group B: 12.8 (9.4)	Group A: 9.6 (2.3) mg/dL Group B: 9.4 (6.9) mg/dL
Lu et al. 2020, Taiwan, Secondary care	Cohort study (12–24 months follow‐up)	1977 American Rheumatism Association preliminary criteria (Wallace et al. 1977)	Tophaceous gout	26	48.4 (9.8)	100	NS	NS	NS	100	NS	NS
McCarthy et al. 1991, United States, setting unclear	Cohort study (10 years follow‐up)	MSU crystal identification or 1977 American Rheumatism Association preliminary criteria (Wallace et al. 1977)	No specified phenotype	Total: 39 Group A—no tophi or radiographic changes of gout: 14 Group B—Radiographic changes of gout, no tophi: 11 Group C—tophaceous gout: 14	**Median (range) at 10‐year follow‐up** Group A: 68 (61–75) Group B: 70 (57–83) Group C: 65 (56–73)	NS	NS	NS	Number (range) in year preceding 10‐year follow‐up: Group A: <1 (0–3) Group B: 2 (0–24) Group C: 2 (0–20) (NB data from 33 participants who continued urate‐lowering therapy for 10 years)	36	**Mean (range) at 10‐year follow‐up** Group A: 18 (10–37) Group B: 27 (12–43) Group C: 28 (13–44)	**At 10‐year follow‐up:** Group A: 6.2 (1.0) mg/dL Group B: 6.3 (1.3) mg/dL Group C: 7.0 (1.8) mg/dL (NB data from 33 participants who continued urate‐lowering therapy for 10 years)
Nehme et al. 2023, United States, population‐based study	Cross‐sectional study	Self‐reported physician diagnosis	No specified phenotype	Total: 2529 Gout: 201 No gout: 2328	Total: 69.2 (6.7) Gout: 69.1 (6.6) No gout: 69.2 (6.7)	**% CI** Total: 46.3 (41.1, 48.5) Gout: 65.8 (56.9, 73.6) No gout: 44.7 (42.3, 47.1)	**% CI** Total: Non‐hispanic white 80.7% (76.9, 84.1) Gout: 81.5% (74.6, 86.9) No gout: 80.7% (76.8, 84.0)	Total: 29.0 (6.3) Gout: 31.4 (7.7) No gout: 28.8 (6.1)	NS	NS	NS	Gout: 6.2 (1.8) mg/dL No gout: 5.6 (1.4) mg/dL
Otter et al. 2020, United Kingdom, podiatry clinic, secondary care	Cross‐sectional study	ACR/EULAR classification criteria (Neogi et al. 2015)	No specified phenotype	Total: 50 Gout: 24 No gout: 26	Gout: 70.63 (11) No gout: 72.73 (10)	Gout: 79 No gout: 81	Gout: Caucasian: 96% Non‐Caucasian: 4% No gout: Caucasian: 96% Non‐Caucasian: 4%	Gout: 30.53 (4.97) No gout: 26.98 (4.77)	Gout flare in last 3 months: Gout 13% No gout N/A	Gout 17% No gout N/A	Gout: 9.39 (8.49) No gout: N/A	NS
Petty et al. 2019, United Kingdom, primary care	Cross‐sectional study	General practitioner diagnosis	No specified phenotype	Total: 128 Gout: 26 No gout: 102	Gout: 66.04 (11.07) No gout: 66.20 (10.75)	Gout: 76.92 No gout: 76.47	Gout White UK/European: 100% Afro Caribbean. Asian/African: 0% No gout White UK/European: 97% Afro Caribbean. Asian/African: 3%	Gout: 30.46 (4.43) No gout: 29.11 (4.71)	NS	NS	NS	NS
Rome et al. 2011, New Zealand, secondary care	Cross‐sectional study	1977 American Rheumatism Association preliminary criteria (Wallace et al. 1977)	No specified phenotype	Total: 50 Gout: 25 Controls: 25	Gout: 61.2 (11.7) Controls: 57.3 (12.2)	Gout: 75 Controls: 75	Gout European Caucasian: 56% Asian: 20% Māori: 20% Pacific: 4% Controls European Caucasian: 60% Asian: 8% Māori: 24% Pacific: 8%	Gout: 32.1 (5.6) Controls: 30.3 (6.4)	Gout: Number in last year: **Mean (SD)** 3.1 (3.7) Controls: N/A	Gout: 52 Controls: N/A	Gout: 22.4 (13.2) Controls: N/A	Gout: 0.40 (0.10) mmol/L Controls: N/A
Rome et al. 2012, New Zealand, secondary care	Prospective observational study (6–8 weeks follow‐up)	1977 American Rheumatism Association preliminary criteria (Wallace et al. 1977)	No specified phenotype	20 (follow‐up 18)	54 (16)	85	New Zealand European: 35% New Zealand Māori: 30% Pacific Islander: 35%	35 (11)	Number in last 3 months: **Mean (SD)** 3.4 (2.7)	45	13.2 (11.4)	0.5 (0.15) mmol/L (follow up 0.42 (0.12))
Stewart et al. 2014, New Zealand, secondary care	Cross‐sectional repeated measures study	1977 American Rheumatism Association preliminary criteria (Wallace et al. 1977)	No specified phenotype	Total: 36 Good footwear group: 21 Poor footwear group: 15	Good footwear group: 57 (13) Poor footwear group: 58 (14)	Good footwear group: 95 Poor footwear group: 87	Good footwear group European: 43% Māori: 19% Pacific: 29% Asian: 10% Poor footwear group European: 40% Māori: 20% Pacific: 20% Asian: 20%	Good footwear group: 35 (8) Poor footwear group: 32 (7)	In last 2 months Good footwear group: 6 (14) Poor footwear group: 1.5 (1.9)	Good footwear group: 71 Poor footwear group: 73	Good footwear group: 13 (8) Poor footwear group: 18 (13)	Good footwear group: 0.41 (0.14) mmol/L Poor footwear group: 0.36 (0.10) mmol/L
Stewart et al. 2015, New Zealand, secondary care	Cross‐sectional observational study	1977 American Rheumatism Association preliminary criteria (Wallace et al. 1977)	No specified phenotype	Total: 87 Gout: 24 Asymptomatic hyperuricaemia: 29 Controls: 34	Gout: 58 (13) Asymptomatic hyperuricaemia: 58 (19) Controls: 58 (14)	Gout: 100 Asymptomatic hyperuricaemia: 100 Controls: 100	Gout European: 58% Māori: 4% Pacific: 21% Asian: 17% Asymptomatic hyperuricaemia European: 83% Māori: 0% Pacific: 10% Asian: 7% Controls European: 88% Māori: 3% Pacific: 0% Asian: 9%	Gout: 30.2 (4.0) Asymptomatic hyperuricaemia: 29.3 (5.9) Controls: 25.0 (2.9)	Gout: **Mean (SD)** 1.3 (1.4) in last 3 months **N (%)** 6 (25%) in 1^st^ MTPJ in last 3 months 21 (88%) history of 1^st^ MTPJ flares	Gout: 71	Gout: 17 (11)	Gout: 0.35 (0.10) mmol/L Asymptomatic hyperuricaemia: 0.46 (0.5) mmol/L Normouricaemic controls: 0.32 (0.6) mmol/L
Stewart, Dalbeth, et al. 2016, New Zealand, secondary care	Cross‐sectional observational study	1977 American Rheumatism Association preliminary criteria (Wallace et al. 1977)	No specified phenotype	Total: 87 Gout: 24 Asymptomatic hyperuricaemia: 29 Normouricaemic controls: 34	Gout: 58 (13) Asymptomatic hyperuricaemia: 58 (19) Normouricaemic controls: 58 (14)	Gout: 100 Asymptomatic hyperuricaemia: 100 Normouricaemic controls: 100	Gout European: 58% Māori: 4% Pacific: 21% Asian: 17% Asymptomatic hyperuricaemia European: 83% Māori: 0% Pacific: 10% Asian: 7% Normouricaemic controls European: 88% Māori: 3% Pacific: 0% Asian: 9%	Gout: 30.2 (4.0) Asymptomatic hyperuricaemia: 29.3 (5.9) Normouricaemic controls: 25.0 (2.9)	Gout: **Mean (SD)** 1.3 (1.4) in last 3 months	Gout: 71	Gout: 17 (11)	Gout: 0.35 (0.10) mmol/L Asymptomatic hyperuricaemia: 0.46 (0.05) mmol/L Normouricaemic controls: 0.32 (0.06) mmol/L
Stewart, Mawston, et al. 2016, New Zealand, secondary care	Two‐arm cross‐sectional study	1977 American Rheumatism Association preliminary criteria (Wallace et al. 1977)	No specified phenotype	Total: 40 Gout: 20 Controls: 20	Gout: 60 (7) Controls: 53 (12)	Gout: 95 Controls: 95	Gout European: 60% Māori: 5% Pacific: 15% Asian: 20% Controls European: 100% Māori: 0% Pacific: 0% Asian: 0%	Gout: 31.5 (5.9) Controls: 26.7 (4.3)	Number in last 3 months: **Mean (SD)** 1.1 (1.6)	Gout: 60	Gout: 16 (11)	Gout: 0.37 (0.15) mmol/L
Stewart, Morpeth, et al. 2016, New Zealand, secondary care	Two‐arm cross‐sectional study	1977 American Rheumatism Association preliminary criteria (Wallace et al. 1977)	No specified phenotype	Total: 40 Gout: 20 Controls: 20	Gout: 60 (7) Controls: 53 (12)	Gout: 95 Controls: 95	Gout European: 60% Māori: 5% Pacific: 15% Asian: 20% Controls European: 100% Māori: 0% Pacific: 0% Asian: 0%	Gout: 31.5 (5.9) Controls: 26.7 (4.3)	Number in last 3 months: **Mean (SD)** 1.1 (1.6)	Gout: 60	Gout: 16 (11)	Gout: 0.37 (0.15) mmol/L
Stewart, Dalbeth, Otter, et al. 2017, New Zealand, secondary care	Cross‐sectional study	1977 American Rheumatism Association preliminary criteria (Wallace et al. 1977)	No specified phenotype	Total: 57 Foot/ankle tophi: 22 No foot/ankle tophi: 35	Foot/ankle tophi: 60 (12) No foot/ankle tophi: 60 (14)	Foot/ankle tophi: 86 No foot/ankle tophi: 89	Foot/ankle tophi European: 27% Māori: 14% Pacific: 59% Asian: 0% No foot/ankle tophi European: 31% Māori: 23% Pacific: 37% Asian: 9%	Foot/ankle tophi: 33 (6) No foot/ankle tophi: 33 (8)	Number in last 3 months: **Mean (SD)** Foot/ankle tophi: 0.5 (0.9) No foot/ankle tophi: 0.6 (1.1)	39	Foot/ankle tophi: 25 (13) No foot/ankle tophi: 11 (10)	Foot/ankle tophi: 0.39 (0.13) mmol/L No foot/ankle tophi: 0.41 (0.13) mmol/L
Stewart, Dalbeth, Vandal, et al. 2017, New Zealand, secondary care	Cross‐sectional study	1977 American Rheumatism Association preliminary criteria (Wallace et al. 1977)	No specified phenotype	Total: 86 Gout: 23 Asymptomatic hyperuricaemia: 29 Normouricaemic controls: 34	Gout: 58 (14) Asymptomatic hyperuricaemia: 58 (19) Normouricaemic controls: 58 (14)	Gout: 100 Asymptomatic hyperuricaemia: 100 Normouricaemic controls: 100	Gout European: 61% Māori: 4% Pacific: 17% Asian: 17% Asymptomatic hyperuricaemia European: 83% Māori: 0% Pacific: 10% Asian: 7% Normouricaemic controls European: 88% Māori: 3% Pacific: 0% Asian: 9%	Gout: 30.8 (3.8) Asymptomatic hyperuricaemia: 29.3 (5.9) Normouricaemic controls: 25.0 (2.9)	Gout: **Mean (SD)** 1.4 (1.4) in last 3 months	Gout: 74	Gout: 18 (11)	Gout: 5.9 (1.7) mg/dL Asymptomatic hyperuricaemia: 7.7 (0.8) mg/dL Normouricaemic controls: 5.4 (1.0) mg/dL
Teh et al. 2013, Malaysia, Secondary care	Cross‐sectional study	1977 American Rheumatism Association preliminary criteria (Wallace et al. 1977)	No specified phenotype	138	56.5 (12.5)	91	Malay: 31.9% Chinese 31.2% Iban 23.2% Bidayuh 10.1% Indian 1.4% Kenyah 0.7% Bisaya 0.7% Punjabi 0.7%	**N (%)** ***Obese*** ^c^ 62 (53.9) ***Overweight*** ^c^ 30 (26.1)	Unclear	47.1	11.6 (8.7)	Pretreatment: 569.6 (142.3) mcg/dL Post‐treatment 421.0 (123.7) mcg/dL
Wang et al. 2015, China, secondary care	Randomised controlled trial	1977 American Rheumatism Association preliminary criteria (Wallace et al. 1977)	Pain in at least one knee joint	60	Surgery + oral medication group: 42.00 (6.91) Oral medication group: 42.57 (8.82)	NS	NS	NS	Surgery + oral medication group: 18 with recurrent attacks, 3–6 episodes per years Oral medication group: 15 with recurrent attacks, 4–6 episodes per year	NS	Surgery + oral medication group: Range 24 hours–5 years Oral medication group: Range 24 hours‐6 years	Surgery + oral medication group: 475.67 (132.37) μmol/L Oral medication group: 468.70 (122.78) μmol/L
Yang et al. 2021, China, secondary care	Cohort study (follow‐up range 6 months to 3 years)	NS	Gout ‘stones’ (tophi) of the hand	55	57 (range 33–81)	89	NS	NS	NS	100	**Mean (range)** 8 (5–18)	**Range** 453–629 μmol/L
Yu et al. 2004, Taiwan, secondary care	Case series	1977 American Rheumatism Association preliminary criteria (Wallace et al. 1977)	Gout and limited knee joint range of motion	7	41.3	100	NS	NS	NS	71	9.7 (5.8)	**Range** 8.5–12.3 mg/dL

Abbreviations: ACR, American College of Rheumatology; BMI, body mass index; CI, confidence interval; EULAR, European Alliance of Associations for Rheumatology; MSU, monosodium urate; MTPJ, metatarsophalangeal joint; N/A, not applicable; NS, not stated; SD, standard deviation.

^a^ Unless otherwise stated.

^b^ Waist circumference ≥ 88 cm in women or ≥ 102 cm in men.

^c^ Not defined.

### 3.3 Description of Impairments

Objectively measured physical impairments are presented in five broad categories: lower extremity function, joint range of motion, strength, deformity and Achilles tendon stiffness (Table 2).

**TABLE 2:** Description of impairments.

Author, year	Physical impairment examined	Method of impairment assessment	Physical impairment
Blandin et al. 2018	Hallux valgus deformity	Plain radiography	***N* (%)** Gout: 67 (62) Controls: 30 (37)
Burke et al. 2015	Lower extremity function Walking speed (m/s) Grip strength (kg)	Short physical performance battery (SPPB) (Guralnik et al. 1994). (0–12, 3 components [standing balance tests, repeat sit to stand, walking speed test], maximum score of 4 for each) 4‐m walk test Dynamometer grip strength	***SPPB*** **Median (25th‐75th percentile)** Gout: 8 (6–10) No gout: 9 (7–10) ***Walking speed*** **Mean (SD)** Gout: 0.85 (0.23) No gout: 0.90 (0.22) ***Grip strength*** **Mean (SD)** Gout: 31.8 (10.9) No gout: 28.8 (10.3)
Carroll et al. 2018	Kinematic and kinetic ankle characteristics: Sagittal plane ankle ROM (°) Frontal plane ankle ROM (°) Peak ankle angular velocity (°/s) Sagittal peak ankle joint force (N) Time to peak ankle joint force (% stance) Peak ankle plantarflexor moment (Nm/kg) Peak ankle plantarflexor moment (% stance) Sagittal peak ankle joint power (W/kg) Ankle plantarflexor concentric work (J/kg)	Three‐dimensional gait analysis using nine‐camera motion analysis system (Qualysis AB, Gothenburg, Sweden) and two floor‐mounted force plates (Advanced medical Technology Inc., USA)	**Mean (SD)** ***Sagittal plane ankle ROM*** Tophaceous gout: 18.0 (3.5) Control: 17.5 (3.1) ***Frontal plane ankle ROM*** Tophaceous gout: 10.4 (3.2) Control: 9.1 (3.7) ***Peak ankle angular velocity*** Tophaceous gout: −210.1 (53.0) Control: −254.5 (42.4) ***Sagittal peak ankle joint force*** Tophaceous gout: 279.3 (70.1) Control: 263.7 (59.9) ***Time to peak ankle joint force*** Tophaceous gout: 80.0 (5.13) Control: 79.3 (7.1) ***Peak ankle plantarflexor moment*** Tophaceous gout: 1.21 (0.21) Control: 1.15 (0.19) ***Peak ankle plantarflexor moment*** Tophaceous gout: 78.5 (1.9) Control: 78.0 (1.6) ***Sagittal peak ankle joint power*** Tophaceous gout: 1.86 (0.68) Control: 2.17 (0.49) ***Ankle plantarflexor concentric work*** Tophaceous gout: 0.16 (0.06) Control: 0.17 (0.04)
Dalbeth et al. 2007	Hand function Finger tip to palm flexion distance (cm) Dominant hand grip strength (kg)	Sollerman hand function test (/80) (Sollerman and Ejeskär 1995) Mean of fingertip to palm flexion distance (distal palmar crease) for all fingers Jamar hand dynamometer (surgical synergies Ltd, NZ)	**Median (range)** ***Sollerman hand function test*** 75.5 (31–80) ***Fingertip to palm flexion distance*** 2.83 (0.78–7.08) ***Grip strength*** 31 (4–71)
Feng and Xiong 2021	Wrist joint range of motion (°)	Unclear	**Mean (SD)** ***Conservative management (n = 13) pre‐treatment*** ***Palmar flexion*** 18.3 (14.5) ***Dorsal extension*** 20.7 (10.5) ***Ulnar deviation*** 11.8 (6.1) ***Radial deviation*** 8.8 (4.1) ***Conservative management (n = 13) 10* *months—9* *years follow‐up (average 2*.*2* *years)*** ***Palmar flexion*** 65.4 (12.1) ***Dorsal extension*** 57.2 (16.4) ***Ulnar deviation*** 21.1 (5.1) ***Radial deviation*** 15.5 (3.8) ***Surgical management (n = 11*, *excluding 3 arthrodesis) pre‐treatment*** ***Palmar flexion*** 11.9 (4.9) ***Dorsal extension*** 8.3 (5.6) ***Ulnar deviation*** 7.9 (3.0) ***Radial deviation*** 7.6 (5.2) ***Surgical management (n = 11) 5* *months—9* *years follow‐up (average 4*.*9* *years)*** ***Palmar flexion*** 49.3 (9.7) ***Dorsal extension*** 41.2 (12.3) ***Ulnar deviation*** 9.4 (8.5) ***Radial deviation*** 18.4 (7.4)
López López et al. 2017	Limited joint motion	Number of joints with limited motion (not defined)	Unclear
Lu et al. 2020	Knee joint range of motion (°)	Goniometer	**Mean (SD)** ***Before urate‐lowering therapy*** 103.5 (5.7) ***After urate‐lowering therapy*** 129.6 (6.5)
McCarthy et al. 1991	Joint deformity	Physical examination for joint malalignment caused by contracture, bony enlargement, bony collapse or subluxation	***Number of joints deformed according to radiographic progression after 10* *years of antihyperuricemic therapy*** Group A (*n* = 14) Reduced: 0 Increased: 1 Unchanged: 0 Group B (*n* = 11) Reduced: 1 Increased: 3 Unchanged: 2 Group C (*n* = 14) Reduced: 0 Increased: 3 Unchanged: 1
Nehme et al. 2023	Hand muscle strength (kg)	Handgrip dynamometer	**Mean (SD)** ***Combined grip strength*** Gout: 66.4 (20.1) No gout: 61.5 (19.9) ***Grip strength right‐hand highest value*** Gout: 33.0 (10.1) Without gout: 30.6 (10.0) ***Grip strength left‐hand highest value*** Gout: 33.4 (10.4) Without gout: 30.9 (10.2)
Otter et al. 2020	Achilles tendon grade Achilles tendon thickness (mm) Achilles tendon stiffness (m/s)	Achilles tendon grade and thickness, ultrasound Achilles tendon stiffness, ultrasound and shear wave elastography	***Achilles tendon* grade, n (%)** **Grade 1—normal appearing tendon with homogeneous fibrillar echotexture** Gout right: 19 (80) Gout left: 17 (71) No gout right: 18 (50) No gout left: 19 (54) **Grade 2—a focal fusiform swelling and/or diffuse enlarged tendon** Gout right: 5 (21) Gout left: 7 (30) No gout right: 8 (31) No gout left: 7 (27) **Grade 3—a hypoechoic area within the tendon with/or without tendon enlargement** Gout right: 0 Gout left: 0 No gout right: 0 No gout left: 0 ***Midpoint Achilles tendon thickness*** Gout right: 5.66 (1.51) Gout left: 5.86 (1.58) No gout right: 5.87 (1.46) No gout left: 5.54 (0.88) ***Achilles tendon stiffness*** Gout right: 8.90 (1.65) Gout left: 9.17 (1.4) No gout right: 9.76 (0.48) No gout left: 9.66 (0.65)
Petty et al. 2019	**Person‐level variables:** Physical performance **Foot level variables:** Toe deformities (MTPJ and interphalangeal joint hyperextension measured at the 1^st^ MTPJ. Hammer, mallet, claw and retracted toe measured at lesser toes) Foot posture 1^st^ MTPJ dorsiflexion (°) Subtalar joint inversion and eversion range of motion (°) Ankle joint range of motion (°)	**Person‐level variables:** Short physical performance battery (SPPB) (Guralnik et al. 1994) (0–12, 3 components [standing balance tests, repeat sit to stand, walking speed test], maximum score of 4 for each) 4‐m walk test (m/s) **Foot level variables:** Toe deformities ‐ physical examination present/absent (Coughlin 2003) Foot posture – Arch index (Cavanagh and Rodgers 1987); navicular height (mm) (Menz and Munteanu 2005); Foot Posture Index‐6 (FPI‐6) (Keenan et al. 2007) Passive 1^st^ MTPJ dorsiflexion non‐weight‐bearing range of motion ‐ Goniometer (Hopson et al. 1995) Subtalar joint inversion and eversion non‐weight‐bearing range of motion ‐ Goniometer (Menadue et al. 2006) Ankle joint weight‐bearing range of motion (knee extended and knee flexed) ‐ Inclinometer (Bennell et al. 1998)	**Mean (95% CI), adjusted for BMI** ***SPPB*** Gout: 8.57 (7.08, 10.05) No gout: 8.08 (6.29, 8.87) **N (%)** ***Hallux valgus*** Gout right foot: 7 (26.92) Gout left foot: 1 (3.85) No gout right: 31 (30.39) No gout left: 29 (28.43) ***Deformities (all toes)*** Gout right foot: 14 (53.85) Gout left foot: 18 (69.23) No gout right: 64 (62.75) No gout left: 61 (59.80) ***1*** ^***st***^ ***MTPJ*** Gout right foot: 3 (11.54) Gout left foot: 1 (3.85) No gout right: 9 (8.82) No gout left: 10 (9.80) ***Hammer (lesser toes)*** Gout right foot: 8 (30.77) Gout left foot: 10 (38.46) No gout right: 31 (30.39) No gout left: 35 (34.31) ***Mallet (lesser toes)*** Gout right foot: 7 (26.92) Gout left foot: 10 (38.46) No gout right: 18 (17.65) No gout left: 10 (9.80) ***Claw (lesser toes)*** Gout right foot: 3 (11.54) Gout left foot: 3 (11.54) No gout right: 23 (22.55) No gout left: 24 (23.53) ***Retracted (lesser toes)*** Gout right foot: 1 (3.85) Gout left foot: 1 (3.85) No gout right: 6 (5.88) No gout left: 7 (6.86) **Mean (95%CI), adjusted for BMI** ***Variables accounting for correlation between feet*** ***Arch index*** Gout: 0.24 (0.22. 0.25) No gout: 0.24 (0.23, 0.25) ***Navicular height*** Gout: 0.17 (0.16, 0.19) No gout: 0.18 (0.17, 0.18) ***Foot posture index*** Gout: 2.20 (1.61, 2.79) No gout: 2.29 (1.95, 2.64) ***1*** ^***st***^ ***MTPJ dorsiflexion*** Gout: 54.42 (47.81, 61.02) No gout: 63.09 (59.56, 66.62) ***Subtalar joint inversion*** Gout: 21.15 (18.23, 24.06) No gout: 26.71 (25.09, 28.34) ***Subtalar joint eversion*** Gout: 10.00 (8.47, 11.52) No gout: 12.12 (11.09, 13.16) ***Ankle joint dorsiflexion—knee extended*** Gout: 62.90 (59.43, 66.37) No gout: 61.94 (60.45, 63.42) ***Ankle joint dorsiflexion—knee flexed*** Gout: 55.03 (51.64, 58.42) No gout: 51.97 (50.32, 53.63)
Rome et al. 2011	Peak plantar pressure (kPa) Pressure time integral (kPa s) Step length (m) Stride length (m) Single leg support (s) Double leg support (s) Stance phase (s) Swing phase (s) Velocity (m/s) Cadence (steps/min)	Plantar pressure measurement: F‐scan mobile system (Tekscan Inc., South Boston, MA, USA) Spatial and temporal parameters of gait: The GAITMAT II	**Mean (SD)** ***Peak plantar pressure*, *left foot*** ***Medial heel*** Gout: 264.9 (98.8) Control: 297.6 (87.2) ***Lateral heel*** Gout: 249.8 (88.2) Control: 272.9 (92.8) ***Midfoot*** Gout: 120.9 (59.3) Control: 119.8 (128.1) ***1*** ^***st***^ ***metatarsal region*** Gout: 252.9 (113.9) Control: 249.6 (140.1) ***2*** ^***nd***^ ***metatarsal region*** Gout: 309.7 (141.6) Control: 281.2 (140.5) ***3*** ^***rd***^ ***metatarsal region*** Gout: 333.3 (173.5) Control: 299.5 (83.7) ***4*** ^***th***^ ***metatarsal region*** Gout: 248.8 (127.1) Control: 246.1 (84.5) ***5*** ^***th***^ ***metatarsal region*** Gout: 153.1 (10.7) Control: 237.8 (112.1) ***1*** ^***st***^ ***toe*** Gout: 143.8 (96.9) Control: 263.8 (123.9) ***2*** ^***nd***^ ***–5*** ^***th***^ ***toes*** Gout: 124.2 (85.9) Control: 213.6 (129.1) ***Peak plantar pressure*, *right foot*** ***Medial heel*** Gout: 253.3 (103.2) Control: 268.2 (83.7) ***Lateral heel*** Gout: 240.4 (88.7) Control: 277.6 (81.8) ***Midfoot*** Gout: 156.4 (74.6) Control: 144.2 (84.4) ***1*** ^***st***^ ***metatarsal region*** Gout: 213.1 (113.5) Control: 243.6 (86.5) ***2*** ^***nd***^ ***metatarsal region*** Gout: 297.5 (121.2) Control: 262.6 (112.1) ***3*** ^***rd***^ ***metatarsal region*** Gout: 313.7 (138.8) Control: 274.5 (94.2) ***4*** ^***th***^ ***metatarsal region*** Gout: 249.7 (103.8) Control: 259.0 (123.9) ***5*** ^***th***^ ***metatarsal region*** Gout: 206.9 (136.4) Control: 255.6 (118.1) ***1*** ^***st***^ ***toe*** Gout: 153.4 (100.1) Control: 248.0 (129.1) ***2*** ^***nd***^ ***–5*** ^***th***^ ***toes*** Gout: 133.4 (70.3) Control: 182.6 (112.7) ***Pressure time integral*, *left foot*** ***Medial heel*** Gout: 53.8 (23.9) Control: 42.1 (13.9) ***Lateral heel*** Gout: 50.8 (19.1) Control: 39.9 (13.3) ***Midfoot*** Gout: 29.9 (10.3) Control: 23.1 (9.7) ***1*** ^***st***^ ***metatarsal region*** Gout: 43.9 (18.5) Control: 43.8 (22.6) ***2*** ^***nd***^ ***metatarsal region*** Gout: 57.0 (22.2) Control: 56.4 (22.9) ***3*** ^***rd***^ ***metatarsal region*** Gout: 65.3 (36.9) Control: 60.2 (19.0) ***4*** ^***th***^ ***metatarsal region*** Gout: 53.8 (25.2) Control: 49.1 (19.5) ***5*** ^***th***^ ***metatarsal region*** Gout: 41.1 (22.8) Control: 42.5 (21.0) ***1*** ^***st***^ ***toe*** Gout: 17.5 (13.0) Control: 33.4 (19.7) ***2*** ^***nd***^ ***–5*** ^***th***^ ***toes*** Gout: 17.5 (13.6) Control: 22.7 (9.6) ***Pressure time integral*, *right foot*** ***Medial heel*** Gout: 46.7 (16.9) Control: 40.6 (14.6) ***Lateral heel*** Gout: 47.6 (15.5) Control: 45.4 (12.5) ***Midfoot*** Gout: 39.1 (21.9) Control: 28.9 (10.9) ***1*** ^***st***^ ***metatarsal region*** Gout: 41.2 (21.8) Control: 36.7 (17.6) ***2*** ^***nd***^ ***metatarsal region*** Gout: 51.1 (21.2) Control: 42.2 (19.1) ***3*** ^***rd***^ ***metatarsal region*** Gout: 59.2 (29.0) Control: 46.9 (23.8) ***4*** ^***th***^ ***metatarsal region*** Gout: 59.5 (27.2) Control: 52.5 (16.5) ***5*** ^***th***^ ***metatarsal region*** Gout: 60.1 (46.8) Control: 49.2 (23.4) ***1*** ^***st***^ ***toe*** Gout: 19.5 (17.0) Control: 32.9 (19.4) ***2*** ^***nd***^ ***–5*** ^***th***^ ***toes*** Gout: 23.7 (16.4) Control: 29.3 (16.8) ***Gait measures*, *left foot*** ***Step length*** Gout: 0.57 (0.1) Control: 0.66 (0.1) ***Stride length*** Gout: 1.14 (0.2) Control: 1.32 (0.2) ***Single leg support*** Gout: 0.41 (0.1) Control: 0.42 (0.1) ***Double leg support*** Gout: 0.19 (0.1) Control: 0.19 (0.1) ***Stance phase*** Gout: 0.99 (0.8) Control: 0.75 (0.1) ***Swing phase*** Gout: 0.48 (0.3) Control: 0.41 (0.1) ***Velocity*** Gout: 1.10 (0.3) Control: 0.90 (0.3) ***Cadence*** Gout: 93.7 (16.9) Control: 113.6 (36.9) ***Gait measures*, *right foot*** ***Step length*** Gout: 0.57 (0.1) Control: 0.66 (0.1) ***Stride length*** Gout: 1.13 (0.3) Control: 1.32 (0.2) ***Single leg support*** Gout: 0.57 (0.8) Control: 0.42 (0.1) ***Double leg support*** Gout: 0.20 (0.1) Control: 0.16 (0.1) ***Stance phase*** Gout: 1.1 (1.2) Control: 0.75 (0.1) ***Swing phase*** Gout: 0.41 (0.1) Control: 0.41 (0.1)
Rome et al. 2012	Forefoot deformity (hallux valgus, MTPJ subluxation, fifth MTPJ exostosis, claw/hammer toes (range 0–12)) Rearfoot deformity (calcaneus valgus/varus angle, ankle range of motion, pes planus/cavus (range 0–7)) Foot posture	Structural Index Score (Platto et al. 1991) Foot Posture Index (Redmond et al. 2008)	**Mean (SD)** ***Structural Index ‐ baseline*** ***Forefoot*** 5 (5) ***Rearfoot*** 6 (3) ***Structural Index ‐ follow‐up*** ***Forefoot*** 5 (5) ***Rearfoot*** 5 (3) ***Foot Posture Index ‐ baseline*** 5 (3) ***Foot Posture Index ‐ follow‐up*** 6 (3)
Stewart et al. 2014	Peak plantar pressure (kPa) Pressure time integral (kPa s) Gait parameters: Velocity (m/s) Step length (m) Stride length (m) Cadence (steps/min)	Plantar pressure measurement: F‐scan mobile system (Tekscan Inc., South Boston, MA, USA) Spatial and temporal parameters of gait: The GAITMAT II	**Own footwear data** ***Peak pressure*** **Mean** ***Medial heel*** 259.0 ***Lateral heel*** 246.4 ***Midfoot*** 184.4 ***Metatarsal 1*** 257.1 ***Metatarsal 2*** 304.1 ***Metatarsal 3*** 317.0 ***Metatarsal 4*** 231.9 ***Metatarsal 5*** 208.5 ***Hallux*** 200.3 ***Lesser toes*** 129.1 ***Pressure time integral*** **Mean** ***Medial heel*** 64.61 ***Lateral heel*** 60.89 ***Midfoot*** 43.94 ***Metatarsal 1*** 50.31 ***Metatarsal 2*** 56.89 ***Metatarsal 3*** 58.77 ***Metatarsal 4*** 55.53 ***Metatarsal 5*** 53.95 ***Hallux*** 30.64 ***Lesser toes*** 21.86 ***Gait parameters*** **Mean** ***Velocity*** 0.852 ***Step length*** 0.566 ***Stride length*** 1.142 ***Cadence*** 90.517
Stewart et al. 2015	1^st^ MTPJ dorsiflexion (°) Isometric muscle force ‐ plantar flexion and dorsiflexion of 1^st^ MTPJ (N) Hallux valgus severity Foot posture	Passive 1^st^ MTPJ dorsiflexion non‐weight‐bearing range of motion ‐ Goniometer (Hopson et al. 1995) CITEC hand‐held dynamometry Hallux valgus ‐ Manchester Scale (Garrow et al. 2001) Foot posture ‐ Foot Posture Index‐6 (FPI‐6) (Redmond et al. 2006)	**Mean** ***1*** ^***st***^ ***MTPJ range of motion*** Gout: 59.7 Asymptomatic hyperuricaemia: 76.8 Controls: 77.6 ***1*** ^***st***^ ***MTPJ Plantar flexion force*** Gout: 71.3 Asymptomatic hyperuricaemia: 114.8 Controls: 92.0 ***1*** ^***st***^ ***MTPJ Dorsiflexion force*** Gout: 58.0 Asymptomatic hyperuricaemia: 65.4 Controls: 57.3 ***Hallux valgus severity*** Odds ratio 0.284 (odds of the diagnostic group moving up one severity category, compared to the control group moving up one severity category (reference grade 0)) ***Foot posture*** Gout: +6.2 Asymptomatic hyperuricaemia: +6.6 Controls: +4.8
Stewart, Dalbeth, et al. 2016	Spatial and temporal parameters of gait Plantar pressure Parameters of gait include: Step length (cm) Stride length (cm) Support base (cm) Step time (s) Swing time (s) Stance time (s) Single support time (s) Double support time (s) Velocity (m/s) Cadence (steps/min)	Spatial and temporal parameters: Barefoot walking on GAITRite system (CIR Systems Inc., New Jersey, US) Dynamic plantar pressure: Barefoot walking on TekScan MatScan system (Boston, MA, USA)	**Least‐squares mean** ***Spatial and temporal gait parameters*, *adjusted for age and BMI*** ***Step length*** Gout: 0.57 Asymptomatic hyperuricaemia: 0.61 Normouricemic control: 0.61 ***Stride length*** Gout: 1.14 Asymptomatic hyperuricaemia: 1.20 Normouricemic control: 1.21 ***Support base*** Gout: 0.10 Asymptomatic hyperuricaemia: 0.11 Normouricemic control: 0.08 ***Step time*** Gout: 0.64 Asymptomatic hyperuricaemia: 0.57 Normouricemic control: 0.60 ***Swing time*** Gout: 0.47 Asymptomatic hyperuricaemia: 0.43 Normouricemic control: 0.46 ***Stance time*** Gout: 0.80 Asymptomatic hyperuricaemia: 0.72 Normouricemic control: 0.74 ***Single support time*** Gout: 0.48 Asymptomatic hyperuricaemia: 0.43 Normouricemic control: 0.46 ***Double support time*** Gout: 0.16 Asymptomatic hyperuricaemia: 0.26 Normouricemic control: 0.16 ***Velocity*** Gout: 0.91 Asymptomatic hyperuricaemia: 1.07 Normouricemic control: 1.03 ***Cadence*** Gout: 95.5 Asymptomatic hyperuricaemia: 107.3 Normouricemic control: 100.9 ***Peak plantar pressure (kPa)*, *adjusted for age and BMI*** ***Heel*** Gout: 268.2 Asymptomatic hyperuricaemia: 301.9 Normouricemic control: 294.3 ***Midfoot*** Gout: 130.8 Asymptomatic hyperuricaemia: 120.1 Normouricemic control: 95.4 ***1*** ^***st***^ ***metatarsal*** Gout: 229.6 Asymptomatic hyperuricaemia: 239.7 Normouricemic control: 211.5 ***2*** ^***nd***^ ***metatarsal*** Gout: 287.1 Asymptomatic hyperuricaemia: 321.3 Normouricemic control: 292.6 ***3*** ^***rd***^ ***–5*** ^***th***^ ***metatarsal*** Gout: 244.1 Asymptomatic hyperuricaemia: 255.2 Normouricemic control: 252.3 ***Hallux*** Gout: 208.4 Asymptomatic hyperuricaemia: 241.9 Normouricemic control: 233.3 ***Lesser toes*** Gout: 121.8 Asymptomatic hyperuricaemia: 107.2 Normouricemic control: 105.9 ***Pressure time integral (kPa s)*, *adjusted for age and BMI*** ***Heel*** Gout: 54.68 Asymptomatic hyperuricaemia: 59.83 Normouricemic control: 61.50 ***Midfoot*** Gout: 32.66 Asymptomatic hyperuricaemia: 27.17 Normouricemic control: 23.48 ***1*** ^***st***^ ***metatarsal*** Gout: 54.24 Asymptomatic hyperuricaemia: 60.25 Normouricemic control: 56.24 ***2*** ^***nd***^ ***metatarsal*** Gout: 70.66 Asymptomatic hyperuricaemia: 82.15 Normouricemic control: 77.61 ***3*** ^***rd***^ ***–5*** ^***th***^ ***metatarsal*** Gout: 61.00 Asymptomatic hyperuricaemia: 64.42 Normouricemic control: 66.61 ***Hallux*** Gout: 34.75 Asymptomatic hyperuricaemia: 41.74 Normouricemic control: 40.66 ***Lesser toes*** Gout: 23.19 Asymptomatic hyperuricaemia: 20.48 Normouricemic control: 21.92
Stewart, Mawston, et al. 2016	Peak isokinetic concentric muscle strength (ankle plantar flexion, dorsiflexion, inversion, eversion). Maximum peak torque Nm/kg normalised to body weight	Biodex System 3 Dynamometer (Biodex medical Systems, Shirley, New York)	**Mean (SD)** ***Plantar flexion (30º/s)*** ***Right*** Gout: 0.52 (0.26) Controls: 0.94 (0.39) ***Left*** Gout: 0.53 (0.25) Controls: 1.02 (0.41) ***Dorsiflexion (30º/s)*** ***Right*** Gout: 0.24 (0.05) Controls: 0.42 (0.09) ***Left*** Gout: 0.26 (0.07) Controls: 0.41 (0.11) ***Plantar flexion (120º/s)*** ***Right*** Gout: 0.32 (0.28) Controls: 0.67 (0.39) ***Left*** Gout: 0.28 (0.16) Controls: 0.56 (0.25) ***Dorsiflexion (120º/s)*** ***Right*** Gout: 0.21 (0.04) Controls: 0.38 (0.15) ***Left*** Gout: 0.21 (0.06) Controls: 0.35 (0.10) ***Eversion (30º/s)*** ***Right*** Gout: 0.23 (0.07) Controls: 0.34 (0.12) ***Left*** Gout: 0.24 (0.08) Controls: 0.33 (0.12) ***Inversion (30º/s)*** ***Right*** Gout: 0.26 (0.11) Controls: 0.36 (0.15) ***Left*** Gout: 0.23 (0.11) Controls: 0.38 (0.17) ***Eversion (120º/s)*** ***Right*** Gout: 0.16 (0.05) Controls: 0.22 (0.07) ***Left*** Gout: 0.16 (0.06) Controls: 0.22 (0.08) ***Inversion (120º/s)*** ***Right*** Gout: 0.17 (0.07) Controls: 0.24 (0.08) ***Left*** Gout: 0.16 (0.07) Controls: 0.25 (0.08)
Stewart, Morpeth, et al. 2016	Spatial and temporal parameters of gait, measured as self‐selected and fast speeds Parameters of gait include: Step time (s) Step length (cm) Stride length (cm) Swing time (s) Stance time (s) Velocity (cm/s) Cadence (steps/min)	Barefoot walking on GAITRite system (CIR Systems Inc, New York, USA)	**Mean** ***Step time*** ***Self‐selected*** Gout: 0.59 Controls:0.54 ***Fast*** Gout: 0.49 Controls: 0.43 ***Step length*** ***Self‐selected*** Gout: 70.4 Controls: 68.1 ***Fast*** Gout: 82.1 Controls: 85.3 ***Stride length*** ***Self‐selected*** Gout: 136.5 Controls: 141.1 ***Fast*** Gout: 164.5 Controls: 170.9 ***Swing time*** ***Self‐selected*** Gout: 0.45 Controls: 0.42 ***Fast*** Gout: 0.40 Controls: 0.36 ***Stance time*** ***Self‐selected*** Gout: 0.72 Controls: 0.65 ***Fast*** Gout: 0.57 Controls: 0.50 ***Velocity*** ***Self‐selected*** Gout: 117.4 Controls: 132.7 ***Fast*** Gout: 171.4 Controls: 201.9 ***Cadence*** ***Self‐selected*** Gout: 103.3 Controls: 113.0 ***Fast*** Gout: 124.5 Controls: 141.2
Stewart, Dalbeth, Otter, et al. 2017	Muscle force Isometric ankle plantarflexion, dorsiflexion, inversion, eversion (N)	Hand‐held dynamometer (CITEC Technics, Haren, Netherlands)	**Least‐squares mean (SD)** ***Plantar flexion force*** Foot/ankle tophi: 75.4 (31.0) No foot/ankle tophi: 99.9 (31.5) ***Dorsiflexion force*** Foot/ankle tophi: 55.7 (18.3) No foot/ankle tophi: 74.2 (28.9) ***Inversion force*** Foot/ankle tophi: 45.5 (19.6) No foot/ankle tophi: 58.2 (20.2) ***Eversion force*** Foot/ankle tophi: 43.4 (18.8) No foot/ankle tophi: 56.2 (19.3)
Stewart, Dalbeth, Vandal, et al. 2017	1^st^ MTPJ dorsiflexion (°) Walking velocity (m/s)	Passive 1^st^ MTPJ dorsiflexion non‐weight‐bearing range of motion ‐ Goniometer (Hopson et al. 1995) Barefoot walking on GAITRite system (CIR Systems Inc., New Jersey, US)	**Mean (SD)** ***1*** ^***st***^ ***MTPJ range of motion*** Gout: 59.0 (19.6) Asymptomatic hyperuricaemia: 76.5 (16.9) Normouricaemic controls: 77.6 (17.4) ***Gait velocity*** Gout: 0.88 (0.17) Asymptomatic hyperuricaemia: 1.05 (0.24) Normouricaemic controls: 1.05 (0.19)
Teh et al. 2013	Joint deformities (unspecified)	NS	**Joint deformity prevalence** 39.1%
Wang et al. 2015	Knee flexion Knee extension	NS	**Mean (SD)** ***Baseline*** ***Knee flexion*** ***Surgery + oral medication*** 92.60 (5.95) ***Oral medication*** 91.97 (5.57) ***Knee extensor*** ***Surgery + oral medication*** 25.03 (3.01) ***Oral medication*** 25.23 (2.74) ***48 weeks*** ***Knee flexion*** ***Surgery + oral medication*** 144.17 (3.73) ***Oral medication*** 145.19 (4.08) ***Knee extensor*** ***Surgery + oral medication*** 4.60 (21.38) ***Oral medication*** 5.00 (1.76)
Yang et al. 2021	Finger deformity Finger function	NS	Numeric data not provided
Yu et al. 2004	Knee joint range of motion (°)	NS	**Range of motion** **Extension and flexion** ***Right*** Case 1: 25–70 Case 2: 0–95 Case 3: 0–135 Case 4: 30–100 Case 5: 0–90 Case 6: 0–135 Case 7: 0–100 ***Left*** Case 1: 30–70 Case 2: 0–100 Case 3: 0–90 Case 4: 30–100 Case 5: 0–90 Case 6: 10–100 Case 7: 0–90

Abbreviations: BMI, body mass index; CI, confidence interval; MTPJ, metatarsophalangeal joint; NS, not stated; ROM, range of motion; SD, standard deviation.

#### 3.3.1 Lower Extremity Function

Using the Short Physical Performance Battery (SPPB) (Guralnik et al. 1994), lower limb function (Kolmogorov‐Smirnov *p* < 0.001) and unadjusted walking speed (*p* < 0.001) were worse in people with gout (*n* = 595) compared to people without gout (*n* = 5224) (Burke et al. 2015).

Several studies undertaken in New Zealand have investigated gait in a number of ways. In a cross‐sectional study of 20 people with gout and 20 controls, spatiotemporal parameters during walking at participant‐selected speed observed greater step time (*p* = 0.017) and stance time (*p* = 0.012), but lower velocity (*p* = 0.031) and cadence (*p* = 0.013) in people with gout than controls. At a faster walking speed, the parameter patterns remained comparable, with the addition of greater swing time among people with gout (*p* = 0.005) (Stewart, Morpeth, et al. 2016). When comparing spatiotemporal parameters and plantar pressure whilst walking barefoot (24 people with gout, 34 age/sex matched controls), step time and stance time were both higher in people with gout (*p* = 0.022, *p* = 0.022, respectively) and velocity was lower (*p* = 0.050). Gout participants also demonstrated lower peak pressures in some foot regions (heel, *p* = 0.012; hallux, *p* = 0.036) and higher pressure in the midfoot (*p* < 0.001), with higher midfoot pressure time integrals (*p* = 0.005) (Stewart, Dalbeth, et al. 2016). In cross‐sectional first metatarsophalangeal joint (MTPJ)‐focused analyses, people with gout had lower plantar flexion force (*p* = 0.012) (*n* = 24 with gout, *n* = 34 age/sex matched controls) (Stewart et al. 2015) and walking velocity (*p* = 0.001) among people with ultrasound‐identified tophus (*n* = 23 with gout, *n* = 34 age/sex matched controls) (Stewart, Dalbeth, Vandal, et al. 2017). Plantar pressure variations among 36 people with gout have also been reported when observing walking in shoes of varying quality (Stewart et al. 2014). In 25 people with chronic gout and 25 age/sex matched controls, people with chronic gout had lower hallux peak plantar pressures (*p* < 0.05) lower hallux pressure time integrals (*p* < 0.05), higher midfoot pressure time integrals (*p* < 0.05), slower walking pace and longer step and stride lengths (Rome et al. 2011).

Similarly, Carroll et al. (2018) compared a range of ankle‐specific kinematic and kinetic parameters in 24 people with gout and 24 controls, but found no difference in gait patterns between groups, except for peak ankle joint angular velocity, which was lower in people with gout (*p* < 0.01) (Carroll et al. 2018).

#### 3.3.2 Joint Range of Motion

A cross‐sectional case‐control comparison (gout, *n* = 24; controls, *n* = 34) observed significantly lower 1^st^ MTPJ range of motion among people with gout (*p* < 0.001) (Stewart et al. 2015). A further analysis in the same cohort comparing 46 gout joints with 68 control joints observed the same trend towards lower 1^st^ MTPJ range of motion among people with gout, but this did not reach statistical significance (Stewart, Dalbeth, Vandal, et al. 2017).

A separate, more recent, cross‐sectional study (gout, *n* = 26; controls, *n* = 102) also reported lower 1^st^ MTPJ range of motion among people with gout (*p* = 0.035), as well as a smaller range of subtalar joint inversion (*p* < 0.001) and eversion (*p* = 0.010) (Petty et al. 2019). Lu et al. (2020) demonstrated that range of motion deficits in the knee of 26 gout participants responded well to ULT over an average of 18.2 months (*p* < 0.001), however full range of motion was not restored (mean 129.6, SD 6.5, following ULT). A small randomised controlled trial comparing arthroscopic debridement and oral medication with oral medication only for gout knee arthritis (*n* = 60) showed that trial participants had knee flexion and extension deficits (Wang et al. 2015). In a case series of seven people with gout, knee flexion deficits were observed in all participants and extension deficits were present in three participants (Yu et al. 2004). In a retrospective cohort study of gout wrist arthritis (*n* = 24), statistically significant improvements in wrist joint range of motion deficits were observed following both conservative and surgical treatment interventions (Feng and Xiong 2021). López López et al. (2017) also observed limited joint motion in people with gout (*n* = 564) but the number of joints involved and extent of restriction were unclear.

#### 3.3.3 Strength

In a cross‐sectional study of foot and ankle strength (*n* = 20 with gout, *n* = 20 matched controls), people with gout had lower plantar flexion, inversion and eversion strength (*p* < 0.05), and plantar flexion/dorsiflexion strength ratio (*p* < 0.05) (Stewart, Mawston, et al. 2016). In the same cohort comparing people with foot or ankle tophi (*n* = 22) with those without (*n* = 35), lower plantar flexion (*p* < 0.001), dorsiflexion (*p* = 0.003), inversion (*p* = 0.003) and eversion (*p* = 0.001) was observed in people with foot or ankle tophi (Stewart, Dalbeth, Otter, et al. 2017). The presence of Achilles tophi also resulted in lower plantar flexion (*p* < 0.001), inversion (*p* = 0.008) and eversion (*p* = 0.001) muscle force (Stewart, Dalbeth, Otter, et al. 2017).

Grip strength was considered by two studies. Burke et al. (2015) (*n* = 595 with gout, *n* = 5224 without gout) observed no difference between grip strength and gout after stratification by sex (females, *p* = 0.08; males, *p* = 0.61). A more recent study by Nehme et al. (2023) (*n* = 201 with gout, *n* = 2328 without gout) observed no association between grip strength and gout (*p* = 0.774) but found a positive association between serum urate and grip strength in people without gout (*p* = 0.028). One study (*n* = 20) found that chronic tophaceous gout was associated with poor hand function (Dalbeth et al. 2007).

In a sample of 55 people with gout who underwent surgery for finger deformity and impaired function, the majority experienced improvement following the procedure; however, the nature of improvement was unclear (Yang et al. 2021).

#### 3.3.4 Deformity

In a cross‐sectional study (*n* = 56 with gout, *n* = 41 controls), people with gout were more likely to have hallux valgus (*p* = 0.0007) (Blandin et al. 2018). Compared to age/sex matched controls (*n* = 34), people with gout (*n* = 24), in a study by Stewart et al. (2015), were more likely to have severe hallux valgus (odds ratio 0.284, 95% confidence interval [CI] 0.085, 0.947, *p* = 0.041). A cross‐sectional study by Petty et al. (2019) observed a higher frequency of mallet toe in the left foot of people with gout (*n* = 26), compared to people without gout (*n* = 102) (*p* < 0.001), but not in the right foot. In an earlier study by McCarthy et al. (1991), radiographic progression was observed in nine out of 39 people with gout over 10‐year follow up, seven of whom had progressive deformity evident on clinical assessment. The description of the specific joints involved was unclear. Similarly, in a sample of 138 people with gout, the presence of joint deformity was common (39%), but details of their nature were unspecified (Teh et al. 2013). In a sample of 20 people experiencing an acute gout flare (*n* = 18 at follow‐up), Rome et al. (2012) observed ‘moderate’ changes in structure at the forefoot and rearfoot, and a pronated foot profile in most participants, all of which showed no significant difference between baseline and 6–8‐week follow‐up when the flare had resolved. Petty et al. (2019) observed no difference in foot posture between gout and non‐gout participants (*p* = 0.791).

#### 3.3.5 Achilles Tendon Stiffness

One study examined Achilles tendon stiffness using ultrasound imaging with shear wave elastography. Compared with age/sex‐matched controls (*n* = 26), people with gout (*n* = 24) had significantly lower Achilles tendon stiffness (right Achilles mean difference 1.04 m/s (95%CI 0.38, 1.7), *p* = 0.003; left Achilles mean difference 0.7 m/s (95%CI 0.09, 1.32), *p* = 0.025) (Otter et al. 2020).

## 4 Discussion

We identified 24 studies describing physical impairments in people with gout. These impairments comprised lower extremity function, joint range of motion, strength, deformity and Achilles tendon stiffness. Since this was a scoping review, we did not assess methodological quality; however, most studies were small and cross‐sectional in nature. Given the predilection of gout for the foot, not surprisingly, most studies assessed impairments in the foot/ankle. Compared with people who do not have gout, we found evidence that people with gout have worse lower extremity function, walk more slowly with longer step and stance times, have lower peak plantar pressures in the hallux and heel and higher plantar pressure and pressure‐time integrals in the midfoot, have reduced range of motion at the 1^st^ MTPJ and subtalar joint, more commonly have hallux valgus, and have less stiff Achilles tendons. They have lower muscle strength in the foot, but two studies found no association between grip strength and gout. Some contrasting or inconsistent observations across included studies may be explained by small sample sizes or differences in objective measurement, for example, different approaches to measurement of lower extremity gait function via gait analysis. In some studies, the method of impairment assessment and outcomes were unclear, indicating variation in study quality.

To our knowledge, this is the first review of physical impairments in people with gout. Limitations of our review methods include not registering our protocol publicly prospectively and not searching grey literature although this is not uncommon for scoping reviews of this nature. As with any systematic or scoping review, our findings are limited by the extent and quality of the published literature. Of the included studies, most were limited by small sample sizes and cross‐sectional design. However, our primary aim was to identify which gout‐related physical impairments have been studied in the existing literature rather than to draw inferences about them. Although we included 24 publications, the study populations of some studies overlapped, and hence further research and new datasets appear warranted.

An important consideration is which people with gout these impairments affect most. Gout clinical phenotypes are broad and include recurrent gout flares, intercritical gout, chronic gouty arthritis, and tophaceous disease (Bursill et al. 2019). It seems likely that people with the most frequent flares, chronic gouty arthritis, or tophi will be most at risk of physical impairments. However, most included studies did not include a specific phenotype or did not specify the phenotype included. Three out of 24 studies recruited only people with tophaceous gout. A cross‐sectional study from New Zealand found that foot and ankle tophi were associated with lower muscle force in the foot and ankle (Stewart, Dalbeth, Otter, et al. 2017). Comorbidities such as cardiovascular disease, chronic kidney disease and osteoarthritis are prevalent in people with gout (Roddy et al. 2007, 2008; Bevis et al. 2018) and can lead to physical impairment and frailty (e.g. Sokhal et al. 2023). However, no studies have considered the impact of comorbidity on physical impairment in people with gout.

Our findings highlight common impairments that could be targeted by physical interventions. Such interventions commonly form part of the management of a variety of other musculoskeletal conditions. In broad terms, lower extremity function, spatiotemporal gait parameters, and their potential modification through rehabilitation are clinically plausible (Charlton et al. 2021). As with all musculoskeletal conditions, restoring and optimising deficits in joint range of motion can improve overall movement and function.

Exercise programmes targeting functional limitation and muscle weakness show effectiveness for osteoarthritis (National Institute for Health and Care Excellence 2022) and other arthropathies (e.g. Alghadir et al. 2019; Azeez et al. 2020; Boudjani et al. 2023; Silva et al. 2023). There have been few studies of exercise interventions for hallux valgus (Hurn et al. 2022). However, a progressive resistance exercise programme has been shown to increase toe flexor strength in older people (Mickle et al. 2016). Toe flexor strength is frequently reduced in people with hallux valgus deformity and is hence, a plausible treatment target (Hurn et al. 2015). A recent pilot and feasibility trial found that a multifaceted, nonsurgical intervention including foot exercises met its predetermined efficacy threshold for improvements in strength of hallux plantar flexion as well as ankle dorsiflexion, plantar flexion, inversion and eversion but not lesser toe plantar flexion, despite adherence to the intervention being impacted by the COVID‐19 pandemic and stay‐at‐home (lockdown) orders (Menz et al. 2023).

A general reduction in Achilles tendon stiffness can occur as a normal part of the ageing process and resistance training has been shown to improve Achilles tendon stiffness in older people (McCrum et al. 2018). In addition to targeted exercise‐based physical interventions, it is plausible that gout‐related physical impairments may also respond positively to foot orthoses interventions (Herchenröder et al. 2021; Hurn et al. 2022).

## 5 Conclusions

We found that existing studies have investigated lower extremity function, joint range of motion, strength, deformity and Achilles tendon stiffness in people with gout, showing that physical impairments appear to be prevalent in people with gout and more common than in those without gout. Our findings suggest that this is a topic worthy of future study and that further research is needed to identify which physical impairments are most relevant to people with gout, investigate their burden in people with gout, and develop novel interventions to target them, including physical interventions such as therapeutic exercise and orthoses.

## Author Contributions

**Pranav Kumar:** design, acquisition of data, analysis, interpretation of data, project administration, writing – original draft, writing – review and editing. **Jenni Buckley:** acquisition of data, analysis, interpretation of data, writing – review and editing. **Edward Roddy:** conceptualisation, design, analysis, interpretation of data, supervision, writing – original draft, writing – review and editing. **Martin J Thomas:** conceptualisation, design, analysis, interpretation of data, supervision, writing – original draft, writing – review and editing. All authors agree to be accountable for all aspects of the work in ensuring that questions related to the accuracy or integrity of any part of the work are appropriately investigated and resolved.

## Ethics Statement

The authors have nothing to report.

## Conflicts of Interest

The authors declare no conflicts of interest.

## Supporting information

Supporting Information S1

## Data Availability

Data sharing is not applicable to this article as no new data were created or analysed in this study.
