# Supplementary material for: Physical Impairments in People With Gout: A Scoping Review
Source: Musculoskeletal Care. 2025 Apr 22;23(2):e70103. doi: 10.1002/msc.70103 (PMC12013536; doi:10.1002/msc.70103)
Supplement: Supplementary file 1 — Supporting Information S1 [file MSC-23-e70103-s001.pdf]

Supplementary information: Database search strategy

Medline (Ovid)

Search no.	Search term
1.	gout*.ti,ab,kf.
2.	toph*.ti,ab,kf.
3.	crystal arth*.ti,ab,kf.
4.	podagra.ti,ab,kf.
5.	exp Gout/
6.	1 or 2 or 3 or 4 or 5
7.	(musc* adj3 (weakness* or strength* or force*)).ti,ab,kf.
8.	proprioception.ti,ab,kf.
9.	gait.ti,ab,kf.
10.	balanc*.ti,ab,kf.
11.	deformit*.ti,ab,kf.
12.	motion.ti,ab,kf.
13.	mov*.ti,ab,kf.
14.	walk*.ti,ab,kf.
15.	(activit* adj3 limitation*).ti,ab,kf.
16.	((joint* or articular) adj3 deformit*).ti,ab,kf.
17.	(physical adj3 (deformit* or impairment*)).ti,ab,kf.
18.	exp Range of Motion, Articular/
19.	exp Proprioception/
20.	exp Muscle Weakness/
21.	exp Gait/
22.	7 or 8 or 9 or 10 or 11 or 12 or 13 or 14 or 15 or 16 or 17 or 18 or 19 or 20 or 21
23.	6 and 22
24.	exp Animals/ not Humans/
25.	23 not 24

AMED (Ovid)

Search no.	Search term
1.	gout*.ti,ab.
2.	toph*.ti,ab.
3.	crystal arth*.ti,ab.
4.	podagra.ti,ab.
5.	exp Gout/
6.	1 or 2 or 3 or 4 or 5
7.	(musc* adj3 (weakness* or strength* or force*)).ti,ab.
8.	proprioception.ti,ab.
9.	gait.ti,ab.
10.	balanc*.ti,ab.
11.	deformit*.ti,ab.
12.	motion.ti,ab.
13.	mov*.ti,ab.
14.	walk*.ti,ab.
15.	(activit* adj3 limitation*).ti,ab.
16.	((joint* or articular) adj3 deformit*).ti,ab.
17.	(physical adj3 (deformit* or impairment*)).ti,ab.
18.	exp Range of Motion/
19.	exp Proprioception/
20.	exp Muscle Weakness/
21.	exp Gait/
22.	7 or 8 or 9 or 10 or 11 or 12 or 13 or 14 or 15 or 16 or 17 or 18 or 19 or 20 or 21
23.	6 and 22
24.	exp Animals/ not Humans/
25.	23 not 24

EMBASE (Ovid)

Search no.	Search term
1.	gout*.ti,ab,kf.
2.	toph*.ti,ab,kf.
3.	crystal arth*.ti,ab,kf.
4.	podagra.ti,ab,kf.
5.	exp Gout/
6.	1 or 2 or 3 or 4 or 5
7.	(musc* adj3 (weakness* or strength* or force*)).ti,ab,kf.
8.	proprioception.ti,ab,kf.
9.	gait.ti,ab,kf.
10.	balanc*.ti,ab,kf.
11.	deformit*.ti,ab,kf.
12.	motion.ti,ab,kf.
13.	mov*.ti,ab,kf.
14.	walk*.ti,ab,kf.
15.	(activit* adj3 limitation*).ti,ab,kf.
16.	((joint* or articular) adj3 deformit*).ti,ab,kf.
17.	(physical adj3 (deformit* or impairment*)).ti,ab,kf.
18.	exp Range of Motion, Articular/
19.	exp Proprioception/
20.	exp Muscle Weakness/
21.	exp Gait/
22.	7 or 8 or 9 or 10 or 11 or 12 or 13 or 14 or 15 or 16 or 17 or 18 or 19 or 20 or 21
23.	6 and 22
24.	exp Animals/ not Humans/
25.	23 not 24

APA PsycInfo (EBSCO)

Search no.	Search term
S1	TI gout* OR AB gout*
S2	TI toph* OR AB toph*
S3	TI “crystal arth*” OR AB “crystal arth*”
S4	TI podagra OR AB podagra
S5	S1 OR S2 OR S3 OR S4
S6	TI (musc* N2 (weakness* or strength* or force*)) OR AB (musc* N2 (weakness* or strength* or force*))
S7	TI proprioception OR AB proprioception
S8	TI gait OR AB gait
S9	TI balanc* OR AB balanc*
S10	TI deformit* OR AB deformit*
S11	TI motion OR AB motion
S12	TI mov* OR AB mov*
S13	TI walk* OR AB walk*
S14	TI (activit* N2 limitation*) OR AB (activit* N2 limitation*)
S15	TI ((joint* OR articular*) N2 deformit*) OR AB ((joint* OR articular*) N2 deformit*)
S16	TI (physical N2 (deformit* or impairment)) OR AB (physical N2 (deformit* or impairment))
S17	DE “Range of Motion”
S18	DE “Proprioception”
S19	DE “Gait”
S20	(S6 OR S7 OR S8 OR S9 OR S10 OR S11 OR S12 OR S13 OR S14 OR S15 OR S16 OR S17 OR S18 OR S19)
S21	S5 AND S20
S22	DE “Animals”
S23	S21 NOT S22

CINAHL Plus with Full Text (EBSCO)

Search no.	Search term
S1	TI gout* OR AB gout*
S2	TI toph* OR AB toph*
S3	TI “crystal arth*” OR AB “crystal arth*”
S4	TI podagra OR AB podagra
S5	(MH “Gout”)
S6	S1 OR S2 OR S3 OR S4 OR S5
S7	TI (musc* N2 (weakness* or strength* or force*)) OR AB (musc* N2 (weakness* or strength* or force*))
S8	TI proprioception OR AB proprioception
S9	TI gait OR AB gait
S10	TI balanc* OR AB balanc*
S11	TI deformit* OR AB deformit*
S12	TI motion OR AB motion
S13	TI mov* OR AB mov*
S14	TI walk* OR AB walk*
S15	TI (activit* N2 limitation*) OR AB (activit* N2 limitation*)
S16	TI ((joint* OR articular) N2 deformit*) OR AB ((joint* OR articular) N2 deformit*)
S17	TI (physical N2 (deformit* or impairment*)) OR AB (physical N2 (deformit* or impairment*))
S18	(MH “Range of Motion”)
S19	(MH “Proprioception”)
S20	(MH “Muscle Weakness”)
S21	(MH “Gait”)
S22	S7 OR S8 OR S9 OR S10 OR S11 OR S12 OR S13 OR S14 OR S15 OR S16 OR S17 OR S18 OR S19 OR S20 OR S21
S23	S6 AND S22
S24	(MH “Animals”) NOT (MH “Human”)
S25	S23 NOT S24
